# Increased expression of LCN2 formed a positive feedback loop with activation of the ERK pathway in human kidney cells during kidney stone formation

**DOI:** 10.1038/s41598-020-75670-w

**Published:** 2020-12-04

**Authors:** Zhang Hui, Zhu Jiang, Du Qiao, Zhao Bo, Kang Qiyuan, Bian Shaohua, Yuan Wenbing, Liu Wei, Luo Cheng, Liu Shuangning, Li Zhengyi, Li Yingyi

**Affiliations:** Department of Urology, Baoji People’s Hospital, Baoji, 721000 Shaanxi China

**Keywords:** Cell signalling, Molecular biology

## Abstract

Kidney stones are a common threat to the health of elderly patients with a high incidence of disease. However, the specific molecular mechanism of the formation of kidney stones has not been elucidated. Here, we combined signalling molecules with signalling pathways in a double positive circulation regulation model. In addition, we found that LCN2 plays a role in promoting kidney stones through regulation of the ERK signalling pathway and expression of other kidney stone-related genes. LCN2 expression was upregulated upon oxalate stimulation. P-ERK1/2 inhibition by U0126 in kidney epithelial cells resulted in decreased expression of LCN2. Furthermore, the upregulation of LCN2 not only depended on the activation of the ERK signalling pathway but also regulated the activation of the ERK signalling pathway. Importantly, upregulation of LCN2 not only caused kidney epithelial cell damage but also promoted the expression of other kidney stone-related genes. Our findings improved the understanding of LCN2 and might lead to the development of new therapeutic and prognostic markers for kidney stones.

## Introduction

Kidney stones are a common threat to the health of elderly patients with a high incidence of disease and are also an important source of urinary system lesions^1^. To date, many studies on kidney stones have suggested that kidney stones have a significant impact on the occurrence and development of kidney cysts, kidney cancer and other kidney diseases; research has also partly revealed that the formation and development of kidney stones and calcium reactive oxygen species (ROS) and other transport metabolism are closely related and has also proven that intracellular IL-2R, p38 and Wnt signalling pathway activation and the occurrence of kidney stones have a potential link^2–4^. However, the specific molecular mechanism of the formation of kidney stones has not been elucidated. Studies have shown that the formation of calcium oxalate stones in the kidney is mostly due to the pathological environment involved in oxalate metabolism^5,6^. Oxalate is a metabolic end product of the body, and it is excreted by the kidneys before glomerular filtration and through kidney tubular two-way transport. Oxalate deposition in the kidney tubules or gaps will develop into kidney stones^7^. Randall plaques (RPs) have a role in promoting the formation of spontaneous calcium oxalate kidney stones. The expression profile of RPs was analysed, and several genes related to RP formation were identified, such as Lipocalin 2 (LCN2)^8^. This also indirectly implies that LCN2 and other genes may be associated with the occurrence and development of kidney stones.

LCN2, also known as neutrophil gelatinase-associated lipocalin (NGAL), is a 25 kDa protein and contains a signal peptide that can be secreted and interacts with matrix metalloproteinase 9 (MMP-9) to form a complex^9^. Like other lipocalin superfamily members, LCN2 participates in many intracellular molecular events, such as the transport of small hydrophobic molecules, prevention of MMP-9 degradation and participation in certain signalling pathways^10^. In addition, studies have shown that LCN2 can promote the migration and proliferation of tumour cells through kinase signalling pathways^11,12^. Although LCN2 has a very important function in tumour cells, some studies have shown that LCN2 is significantly overexpressed in kidney-related tissues and changes in LCN2 expression can be used as sensitive and specific kidney function tests. However, so far, there is no research to clarify the relationship between LCN2 and the formation and development of kidney stones and to reveal its specific molecular mechanism and mode of action.

In summary, kidney stones, as a common kidney disease, have a serious impact on the health and quality of life of patients, and coupled with late-occurring kidney damage and complications, kidney stones attracted our attention. This is also the significance of this study. The aim of this study was to identify differentially expressed genes related to the formation of kidney stones by screening a clinical sample dataset. Based on existing studies, we attempted to elucidate the mechanism of the formation and development of kidney stones from the classical kinase pathway to provide a theoretical basis for the prevention and treatment of kidney stones.

## Materials and methods

### Cells and cell culture

HK-2 and HEK293 cells were obtained from the Cell Biology Institute of the Chinese Academy of Sciences (Shanghai, China). The HK-2 and HEK293 cells were cultured in Calcium-free Dulbecco’s modified Eagle’s medium (DMEM) containing 10% (v/v) fetal bovine serum (FBS) (Gibco, Thermo Scientific, Grand Island, NY, USA) in a humidified incubator at 37 °C with 5% CO_2_. For the construction of the cell model, the medium needs to be replaced with a calcium-free DMEM medium before the oxalate treatment.

### Plasmids and siRNAs

The full-length sequence of LCN2 was cloned from cDNA that was reverse transcribed from total cellular RNA into the pCDNA3.1 vector (Invitrogen, Shanghai, China). The cells are cultured in a culture plate, and when they are grown to 80–90%, plasmid transfection can be performed. Transient transfection of cells requires the use of Lipofectamine 2000 (Invitrogen, Carlsbad, CA, USA), and the protocol is performed according to standard instructions.

Oligonucleotides were chemically synthesized and purified by GenePharma (Shanghai, China). The small interfering RNA (siRNA) duplexes used were LCN2, #1, 5′-UGGGCAACAUUAAGAGUUAUU-3′ (sense) and 5′-UAACUCUUAAUGUUGCCCAUU-3′ (antisense), #2, 5′-GAGCUGACUUCGGAACUAAUU-3′ (sense) and 5′-UUAGUUCCGAAGUCAGCUCUU-3′ (antisense) and #3, 5′-GAAGACAAAGACCCGCAAAUU-3′ (sense) and 5′-UUUGCGGGUCUUUGUCUUCUU-3′ (antisense). These three kinds of siRNAs were mixed (siRNA pool) to achieve the interference effect while eliminating the off-target effect by reducing the use of each kind of siRNA. The small interfering RNA (siRNA) duplexes used as negative controls were 5′-UUCUCCGAACGUGUCACGTCACG-3′ (sense) and rev, 5′-ACGUGACACGUUCGGAGAGCTTT-3′ (antisense). Cells were transient transfected with siRNA requires the use of Lipofectamine 2000, and the protocol is performed according to standard instructions.

### Reagents and antibodies

Recombinant human LCN2 (rhLCN2), U0126 and LY294002 were purchased from R&D systems (Minneapolis, MN, USA). LCN2, AKT, phospho-AKT and GAPDH antibodies were purchased from Cell Signaling Technology (Danvers, MA, USA). Phospho-ERK1/2 antibody was purchased from Santa Cruz Biotechnology (Santa Cruz, CA, USA). ERK1/2 antibody was purchased from Merck Millipore (Merck Millipore, MA, USA). GPX3 and MMD antibodies were purchased from Abcam (Abcam, Cambridge, UK). HRP-conjugated secondary antibodies were purchased from Santa Cruz Biotechnology.

### Western blotting analysis

Cells were lysed with cell lysis buffer (20 mM Tris PH7.5, 150 mM NaCl, 1% Triton X-100, 2.5 mM sodium pyrophosphate, 1 mM EDTA, 1% Na3VO4, 0.5 μg/ml leupeptin, 1 mM PMSF). Briefly, the total protein extract was subjected to SDS-PAGE gel electrophoresis and transferred to nitrocellulose (NC) membrane. Finally, the membrane after blocking with 5% skim milk powder is incubated with a specific antibody solution for the membrane of the target molecular weight region. The specific protein band can then be incubated with a HRP-conjugated secondary antibody that matches the primary antibody for 1 h at roomtemperature and visualized with electrochemiluminescence (ECL) reagent (Thermo Scientific, Grand Island, NY, USA) by ImageQuant LA4000mini imaging system (GE Healthcare). Densitometry analysis was performed using ImageJ software, and band intensities were normalized to internal reference.

### RT-PCR

Total RNA extracted from cultured human kidney epithelial cell lines were reverse transcribed with Oligo dT (Promega, WI, USA) according to the manufacturer’s instructions. And the reverse transcribed material was amplified with Taq DNA polymerase (Promega, WI, USA) using a primer pair specific to LCN2 (forward primer, CCACCTCAGACCTGATCCCA; reverse primer, CCCCTGGAATTGGTTGTCCTG), PTGS1 (forward primer, CGCCAGTGAATCCCTGTTGTT; reverse primer, AAGGTGGCATTGACAAACTCC), GPX3 (forward primer, AGAGCCGGGGACAAGAGAA; reverse primer, ATTTGCCAGCATACTGCTTGA), MMD (forward primer, CATCGGCTGTCTGATGACTG; reverse primer, CAGGGGTCCAAGTTCACGAA), GAPDH (forward primer, GGAGCGAGATCCCTCCAAAAT; reverse primer, GGCTGTTGTCATACTTCTCATGG). For PCR, 40 cycles were used at 95 °C for 15 s, 60 °C for 30 s, and 72 °C for 30 min.

### Cell viability assay

The resuspended cell suspension was counted, and then seeded in a 96-well plate at a density of 5000 cells/well in a constant temperature incubator for 24 h. Cell viability was measured using Cell Counting Kit-8 (Dojindo Molecular Technologies, Japan) according to the instructions. The solution absorbance value after the end of the reaction was measured using a Multi-Mode Microplate Reader (PerkinElmer, Finland).

### GEO public online database

The Gene Expression Omnibus (GEO) were used to analyze the expressions of kidney stones related genes in clinical kidney stones patients. GSE73680 datasets contains kidney papillary RP and non-RP tissues of 23 CaOx stone formers (SFs) and normal papillary tissue of 7 controls.

### Statistical analysis

Data were presented as mean ± standard error of the mean (SD) which represent mean of at least three independent experiments. Statistical analyses were performed using GraphPad Prism 5.0 software (GraphPad Software, USA). Student’s t-test was used for comparison between two groups, and analysis of variance was used for comparison among groups. Values of P < 0.05 were considered statistically significant. All experiments were repeated at least three times.

## Results

### LCN2 is highly expressed in patients with kidney stones

**Figure 1:**
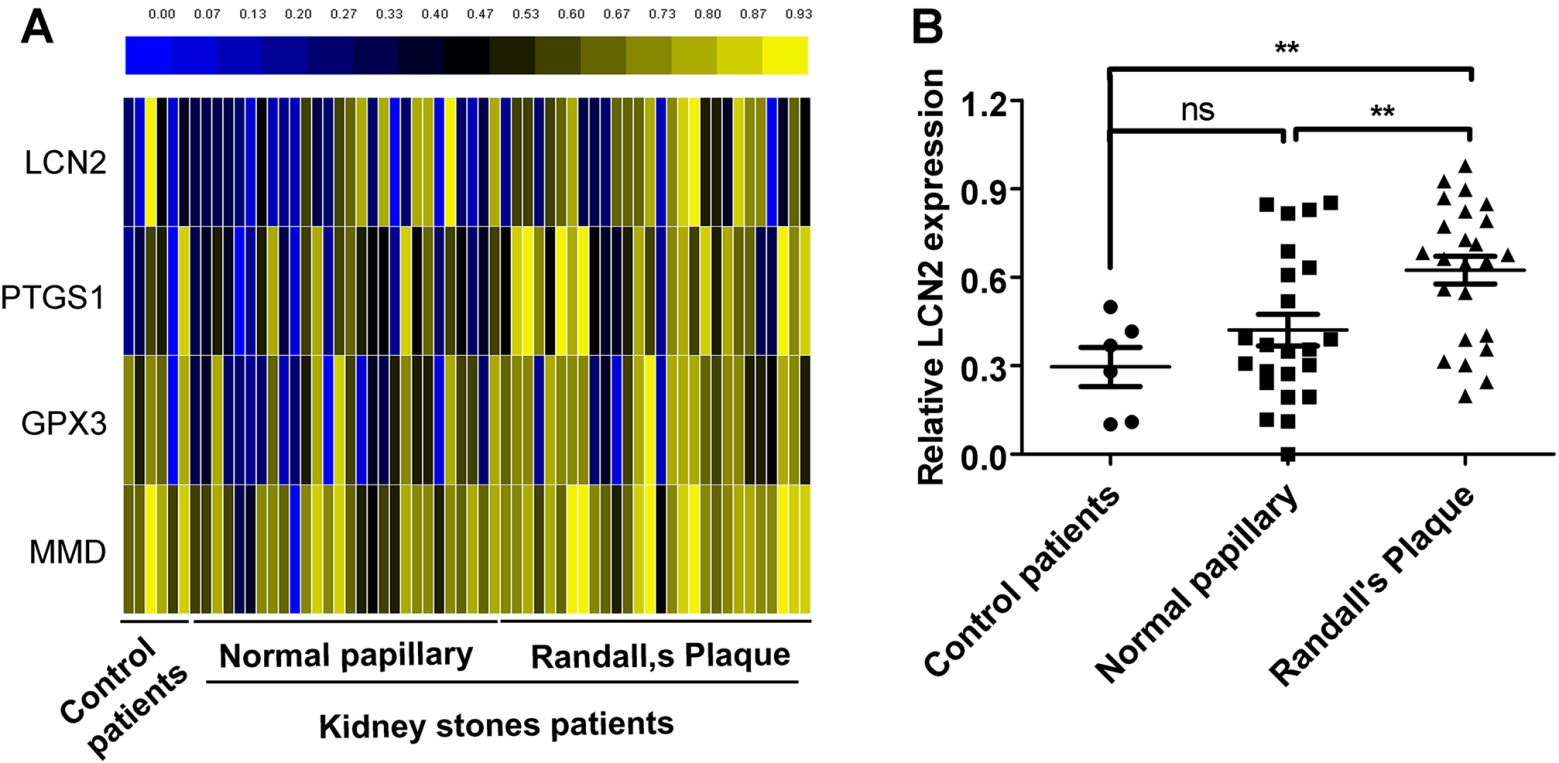
LCN2 is highly expressed in patients with kidney stones. (A and B)GEO datasets mining analysis of LCN2, PTGS1, GPX3 and MMD mRNA levels in GSE73680 dataset between RPs tissue and normal papillary tissue and non-RP tissues of CaOx stone formers. (**P < 0.01).

Some previous studies have shown that LCN2, PTGS1, GPX3 and MMD may play a role in the development and progression of kidney stones. To determine the role of LCN2 in the course of occurrence and development of kidney stones, GEO datasets were analysed. The results showed that LCN2, PTGS1, GPX3 and MMD mRNA expression was increased in Randall plaque (RP) tissue compared with that in normal papillary tissue and non-RP tissues of CaOx stone-formers (Fig. 1A). In particular, LCN2 showed significantly high expression in RP tissue (Fig. 1B). These results are also consistent with previous studies^8^, indicating that LCN2 may play an important role in the occurrence and development of kidney stones.

### Oxalate induced high expression of LCN2 in kidney epithelial cells

**Figure 2:**
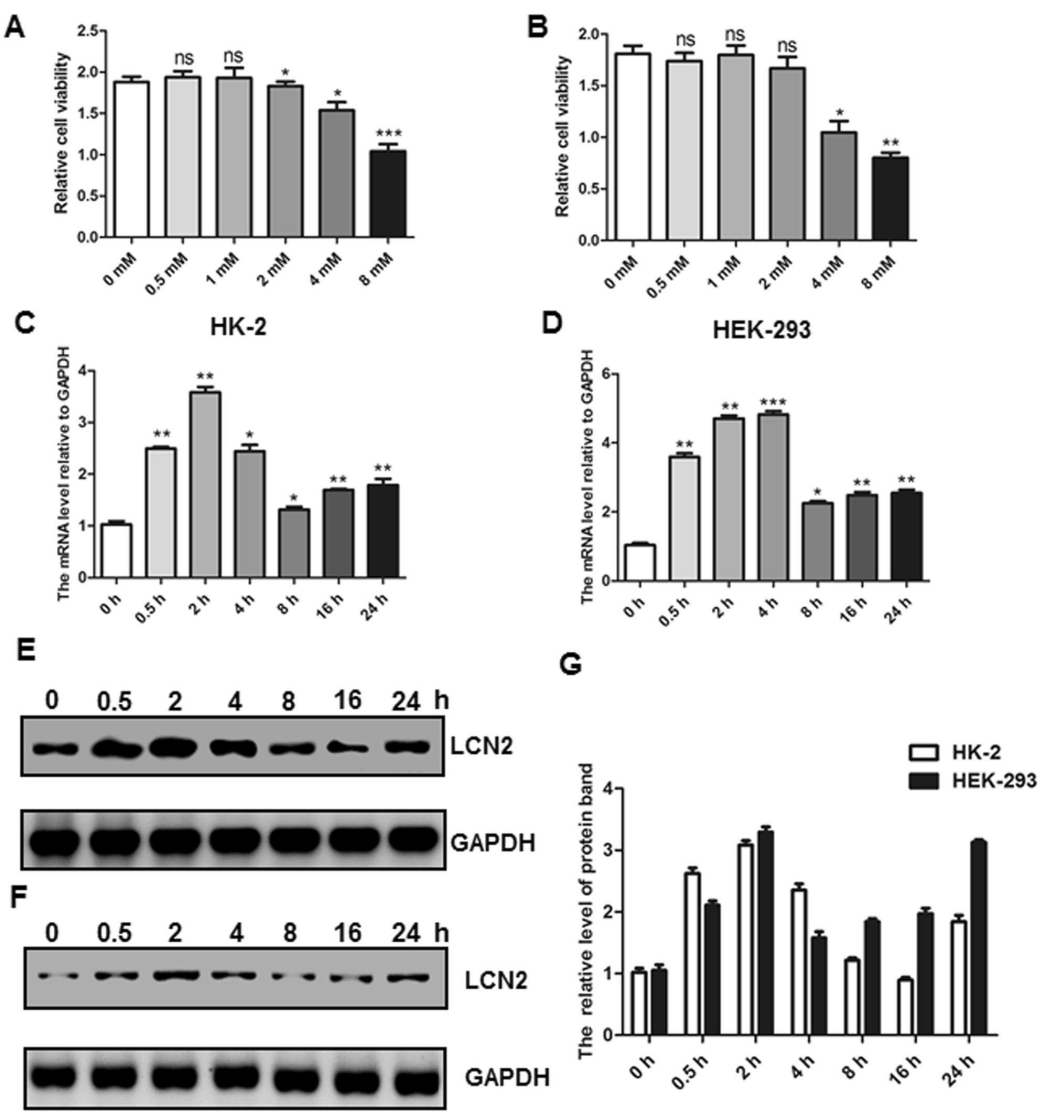
Oxalate induced high expression of LCN2 in renal epithelial cells. HK-2 (**A**) and HEK-293 (**B**) were treated with indicated concentrations of oxalate. The cell viability was determined and analyzed. HK-2 (**C**) and HEK-293 (**D**) were treated with 1 mM oxalate for indicated time. The expression of LCN2 was analyzed by RT-PCR. HK-2 (**E**) and HEK-293 (**F**) were treated with 1 mM oxalate for indicated time. The expressions of LCN2 and GAPDH were analyzed by western blot. (**G**) The quantitative results of bands in the E and F. All experiments were repeated at least three times. (*P < 0.05, **P < 0.01, ***P < 0.001).

To further study the role of LCN2 and the specific molecular mechanisms, we examined changes in kidney epithelial cells (HK-2 and HEK-293) upon oxalate stimulation. First, we examined the effect of oxalate stimulation on cell viability. The results showed that there was no significant change in the cell activity of HK-2 cells stimulated with 1 mM oxalate. When the concentration was greater than 1 mM, the cell viability was significantly decreased (Fig. 2A). Similarly, for HEK-293 cells, the critical concentration of oxalate stimulation was 2 mM (Fig. 2B). Therefore, to ensure normal cellular activity, we treated different cells using their corresponding critical stimulatory concentrations. With this treatment, we demonstrated that the LCN2 mRNA levels were significantly increased by RT-PCR. In particular, the LCN2 mRNA levels reached the highest peak at 2 h (HK-2) or 4 h (HEK-293), and the expression levels decreased with time (Fig. 2C–D). Furthermore, to determine the change in this expression, we further examined the protein level of LCN2 upon oxalate treatment, and the results were consistent with the trend for mRNA. There was a significant increase in the expression of LCN2 in HK-2 and HEK293 cells (Fig. 2E–G). These results indicate that oxalate treatment can induce an increase in the expression of LCN2 in kidney epithelial cells, suggesting that LCN2 may play an important role in oxalate-induced kidney stones.

### The effect of LCN2 on the function of kidney epithelial cells

**Figure 3:**
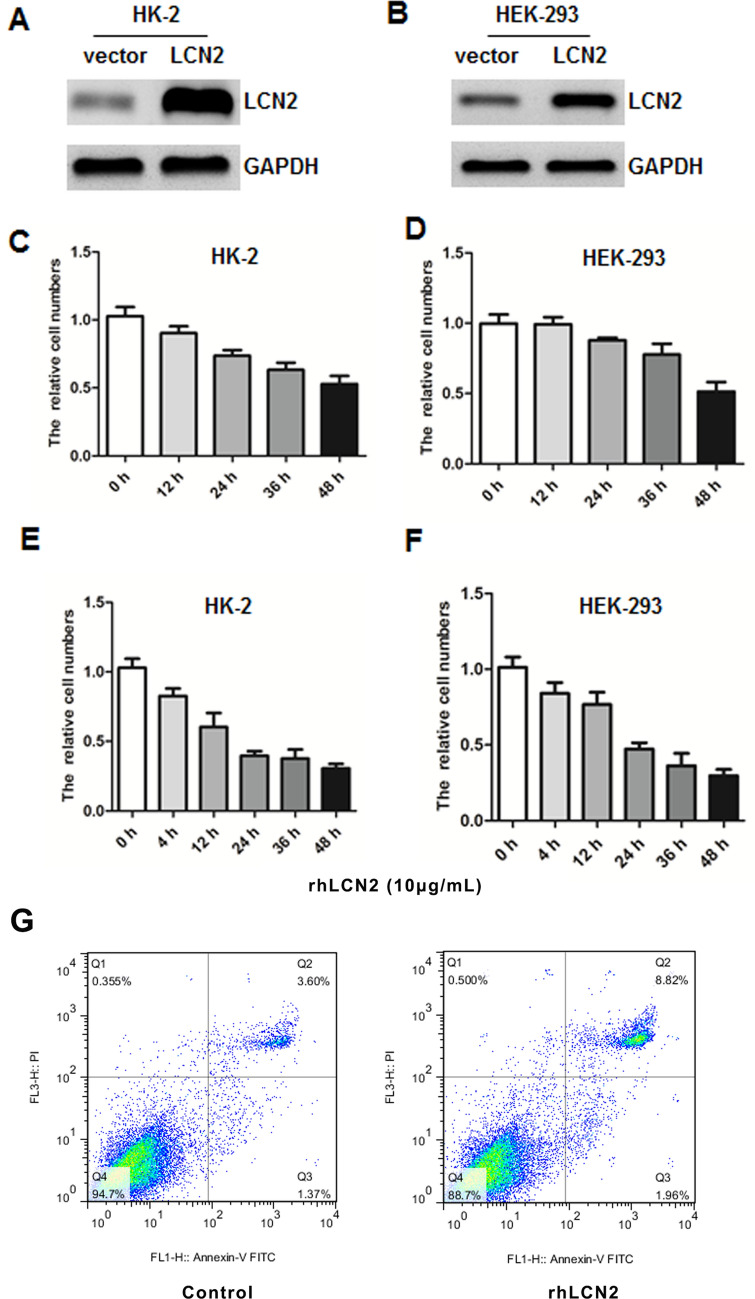
LCN2 affects the renal function. HK-2 (**A**) and HEK-293 (**B**) were transfected with pCDNA3.1 vector or LCN2, and the expressions of LCN2 and GAPDH were analyzed by western blot. HK-2 (**C**) and HEK-293 (**D**) were transfected with pCDNA3.1 vector or LCN2, and then examined the number of cells at each time point. HK-2 (**E**) and HEK-293 (**F**) were treated with rhLCN2, then examined the number of cells at each time point. All experiments were repeated at least three times. (*P < 0.05, **P < 0.01, ***P < 0.001).

To further explore the role of LCN2 in the formation of kidney stones, we constructed an overexpression plasmid. The results of western blotting showed that overexpression of LCN2 could significantly increase the expression of LCN2 in cells (Fig. 3A–B). Studies have shown that kidney stones can cause kidney epithelial cells to undergo apoptosis. Therefore, we used kidney epithelial cell damage to reflect the symptoms of kidney stones at the cellular level. We overexpressed LCN2 in HK-2 and HEK-293 kidney epithelial cells and then examined the number of cells at each time point. The results showed that the number of cells decreased significantly over time, especially after 24 h of overexpression, and the cells showed a significant increase in apoptosis (Fig. 3C–D). Moreover, because LCN2 is a secreted protein that functions by interacting with a specific receptor on the surface of the cell membrane, we used recombinant human LCN2 (rhLCN2) to stimulate HK-2 and HEK-293 cells with results consistent with overexpression of LCN2. With prolongation of the stimulation time, the number of cells showed a significant decrease (Fig. 3E–F). These results suggest that upregulation of LCN2 can cause increased apoptosis of kidney epithelial cells, indicating a certain degree of symptoms of kidney stones.

### The upregulation of LCN2 is dependent on ERK1/2 signalling pathways

**Figure 4:**
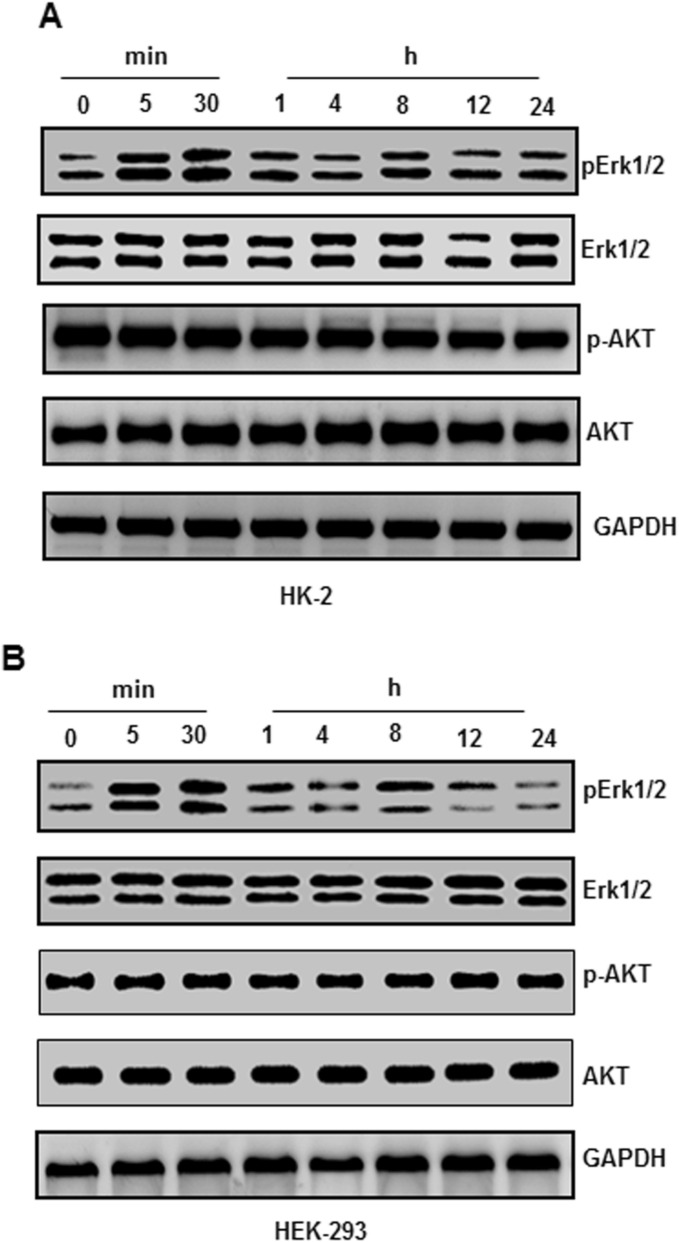
The upregulation of LCN2 is dependent on ERK1/2 signaling pathways. HK-2 (**A**) and HEK-293 (**B**) were treated with oxalate. The expressions of p-ERK1/2, ERK1/2, p-AKT, AKT and GAPDH were analyzed by western blot. All experiments were repeated at least three times.

**Figure 5:**
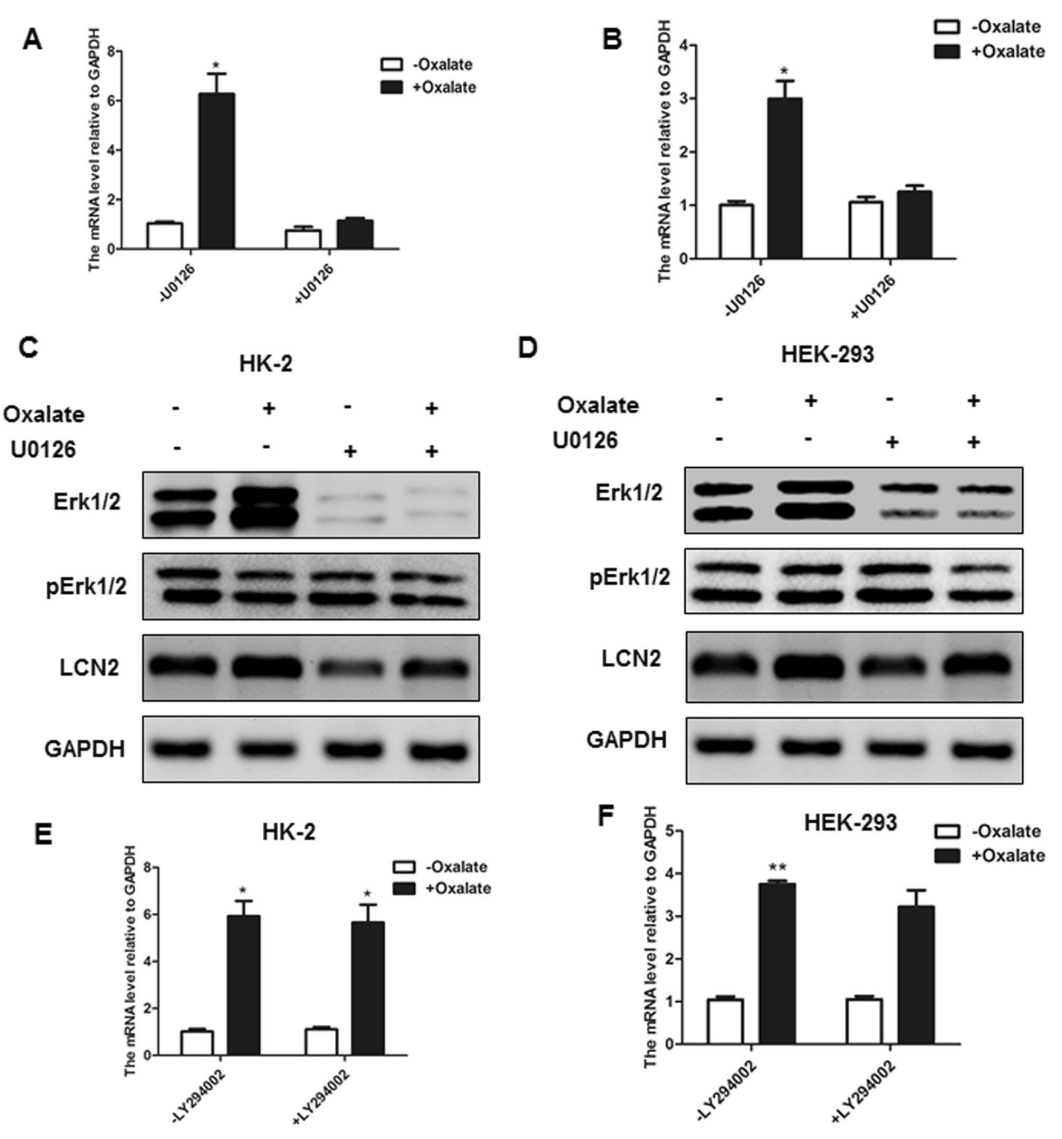
The effect of LCN2 on renal function is dependent on ERK1/2 signaling pathways. HK-2 (**A**) and HEK-293 (**B**) were treated with or without 1 mM oxalate or U0126. The expression of LCN2 was analyzed by RT-PCR. HK-2 (**C**) and HEK-293 (**D**) were treated with 1 mM oxalate for indicated time. The expressions of p-ERK1/2, ERK1/2, LCN2 and GAPDH were analyzed by western blot. HK-2 (**E**) and HEK-293 (**F**) were treated with or without 1 mM oxalate or LY294002. The expression of LCN2 was analyzed by RT-PCR. All experiments were repeated at least three times. (*P < 0.05, **P < 0.01).

We investigated the changes in ERK and AKT signalling pathways in the HK-2 and HEK-293 cell lines to further study the specific molecular mechanism of LCN2 on kidney Fig. 4 stones. The results showed that the protein levels of p-ERK1/2 significantly increased in a short time (5–30 min) upon oxalate stimulation, and then p-ERK1/2 decreased with prolonged stimulation (Fig. 4A–B). Unlike p-ERK1/2, the expression level of p-AKT protein was relatively constant, and there was no significant change before or after oxalate stimulation (Fig. 4A–B). To further study the relationship between LCN2 and the ERK signalling pathway under the stimulation of oxalate, we used the inhibitor U0126 to inhibit the activity of the ERK signalling pathway and detected the expression of LCN2. The results showed that when the inhibitor U0126 was not added, stimulation significantly increased the mRNA levels of LCN2, while the use of U0126 to a large extent eliminated this change (Fig. 5A–B). We also examined changes in LCN2 protein levels in the same treatment groups, and the results were consistent with the changes in mRNA levels (Fig. 5C–D). When the inhibitor LY294002 was used, the AKT signalling pathway did not completely eliminate the effect of oxalate on the increase in LCN2 expression (Fig. 5E–F). These results suggest that oxalate regulates the expression of LCN2 through the ERK signalling pathway and not the AKT signalling pathway.

### The level of p-ERK1/2 was affected by LCN2

**Figure 6:**
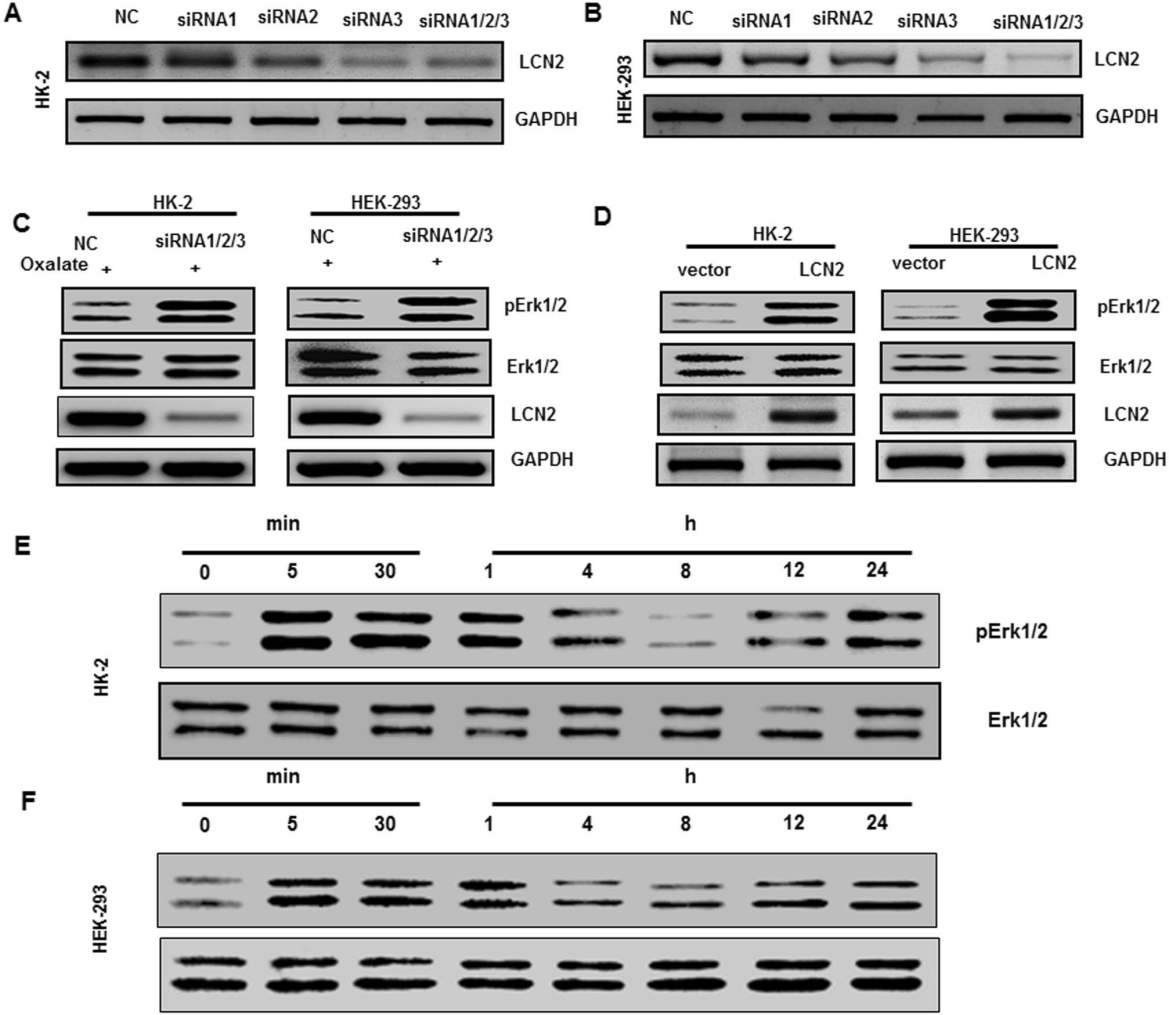
The level of pERK1/2 was affected by LCN2. HK-2 (**A**) and HEK-293 (**B**) were transfected with negative control (NC) siRNA or LCN2 siRNA 1/2/3, and the expressions of LCN2 and GAPDH were analyzed by western blot. (**C**) HK-2 and HEK-293 were transfected with NC siRNA or LCN2 siRNA1/2/3 pools, and the expressions of p-ERK1/2, ERK1/2, LCN2 and GAPDH were analyzed by western blot. (**D**) HK-2 and HEK-293 were transfected with vector or LCN2, and the expressions of p-ERK1/2, ERK1/2, LCN2 and GAPDH were analyzed by western blot. HK-2 (**E**) and HEK-293 (**F**) were treated with rhLCN2, and the expressions of p-ERK1/2 and ERK1/2 were analyzed by western blot. All experiments were repeated at least three times.

In this study, we demonstrated that the ERK signalling pathway regulates the expression of LCN2. Therefore, to reveal the relationship between them, we further investigated the effect of LCN2 on the activity of the ERK signalling pathway. We successfully knocked down the expression of LCN2 in cells by transient transfection with siRNA pools (Fig. 6A–B). Based on this, we examined the expression of p-ERK upon oxalate stimulation. The results showed that knockdown of LCN2 in the HK-2 and HEK-293 cell lines reduced the expression of p-ERK or prevented the increase in p-ERK expression upon oxalate stimulation (Fig. 6C). In contrast, overexpression of LCN2 significantly increased the expression of p-ERK (Fig. 6D). Because of this, we treated kidney epithelial cells with rhLCN2. The results showed that short-term stimulation could activate the intracellular ERK signalling pathway, and then the activity level fluctuated over time (Fig. 6E–F). These results suggest that although the ERK signalling pathway could regulate LCN2 expression, the activity of its own signalling pathway is also affected by LCN2. This constitutes positive feedback regulation.

### LCN2 promotes the expression of other proteins associated with kidney stones through ERK1/2

**Figure 7:**
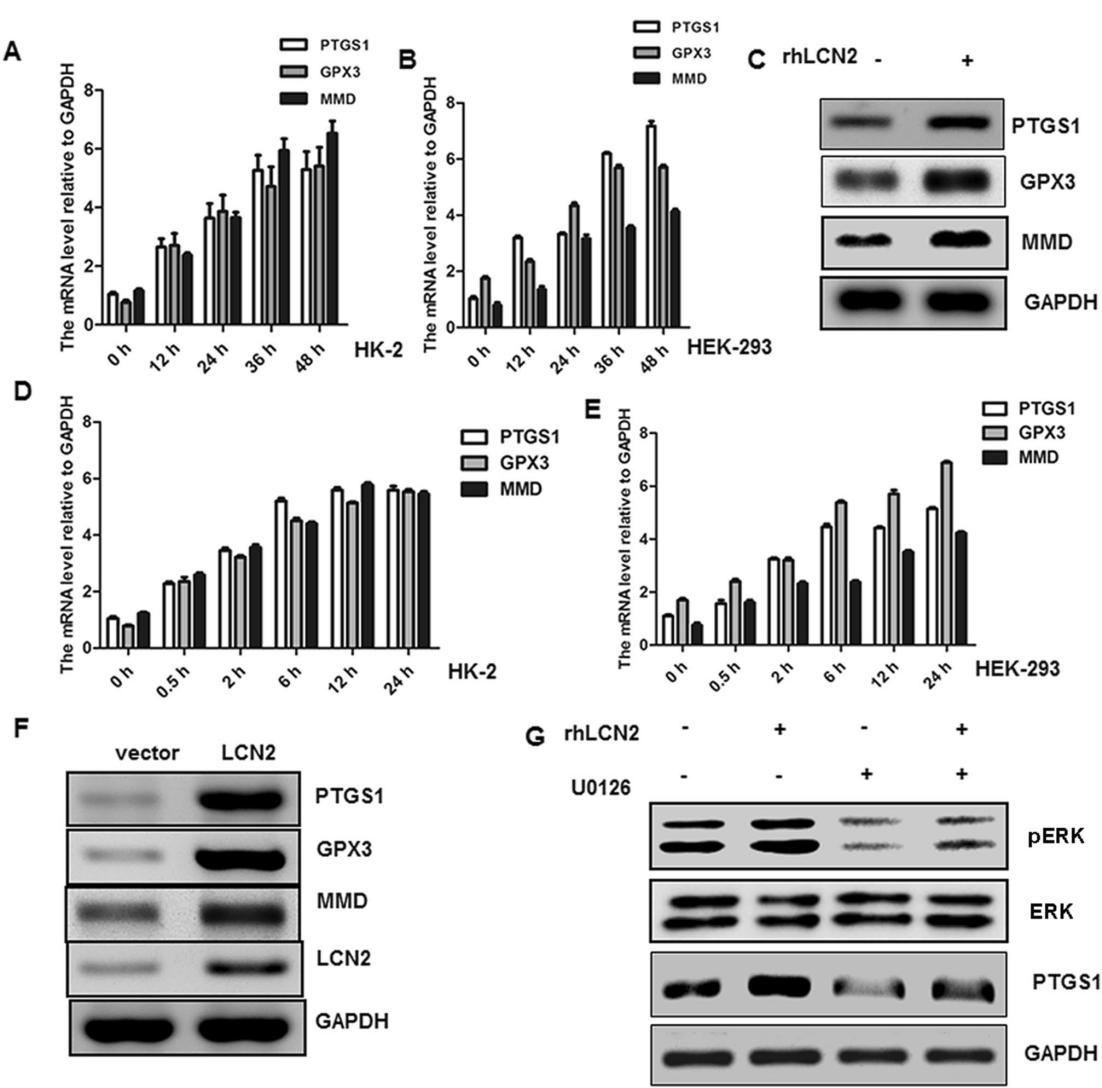
LCN2 promotes the expression of other proteins associated with kidney stones through ERK1/2. HK-2 (**A**) and HEK-293 (**B**) were transfected with vector or LCN2 for indicated time, and the expressions of PTGS1, GPX3 and MMD were analyzed by RT-PCR. (**C**) HK-2 was treated with rhLCN2, and the expressions of PTGS1, GPX3 and MMD were analyzed by western blot. HK-2 (**D**) and HEK-293 (**E**) were treated with rhLCN2, and the expressions of PTGS1, GPX3 and MMD were analyzed by western blot. (**F**) HK-2 was transfected with vector or LCN2 for 36 h, the expressions of PTGS1, GPX3 and MMD were analyzed by western blot. (G) HK-2 was treated with or without rhLCN2 or U0126, and the expressions of p-ERK1/2, ERK1/2, PTGS1 and GAPDH were analyzed by western blot. All experiments were repeated at least three times.

Based on the results of the data set analysis, PTGS1, GPX3 and MMD were also highly expressed in the samples of kidney stones. Similarly, given the results of previous studies, these molecules do have a close relationship with the development of kidney stones^8^. Therefore, we also examined the effect of LCN2 on the expression of other kidney stone-related genes. The results showed that overexpression of LCN2 increased the expression of PTGS1, GPX3 and MMD mRNA (Fig. 7A–B) and protein (Fig. 7F) in cells. Similarly, treatment of kidney epithelial cells with rhLCN2 also increased the expression of PTGS1, GPX3 and MMD mRNA (Fig. 7D–E) and protein (Fig. 7C) in cells. However, this trend was significantly reduced in U0126 inhibitor-treated cells and the effect of rhLCN2 treatment on the increased expression of PTGS1 was significantly inhibited.

## Discussion

Kidney stones are a common disease in urology, mostly occurring in men in the prime of life, and can cause kidney colic, haematuria and other symptoms; severe cases can cause kidney infection or obstruction, causing water and lumps and ultimately affecting kidney function^13,14^. The formation mechanism of kidney stones has not yet been elucidated. Kidney epithelial cell damage is considered to be an important cause of the formation of oxalate stones. Damaged cells not only provide effective sites for the formation of initial nuclei and promote the formation of early micro-stones but also enhance membrane and mineral microcrystalline adhesion, accelerating the formation of kidney stones. The debris after cell damage can also provide conditions for oxalate heterozygous nucleation^15^. In view of the molecular mechanism of the formation of kidney stones, studies have shown that LCN2 may play an important role^16^. LCN2, which is also known as neutrophil gelatinase-associated LCN, is expressed in tubular cells and neutrophils and is related to cellular apoptosis and inflammation^17,18^. However, little is known about the role of LCN2 in kidney stones. There are also studies that indicate that LCN2 can be regulated and that it is associated with many signalling pathways in certain tumour cell models^10–19^. However, little is known about the specific regulation of LCN2 and the role of LCN2 and related signalling pathways in kidney stones.

**Figure 8:**
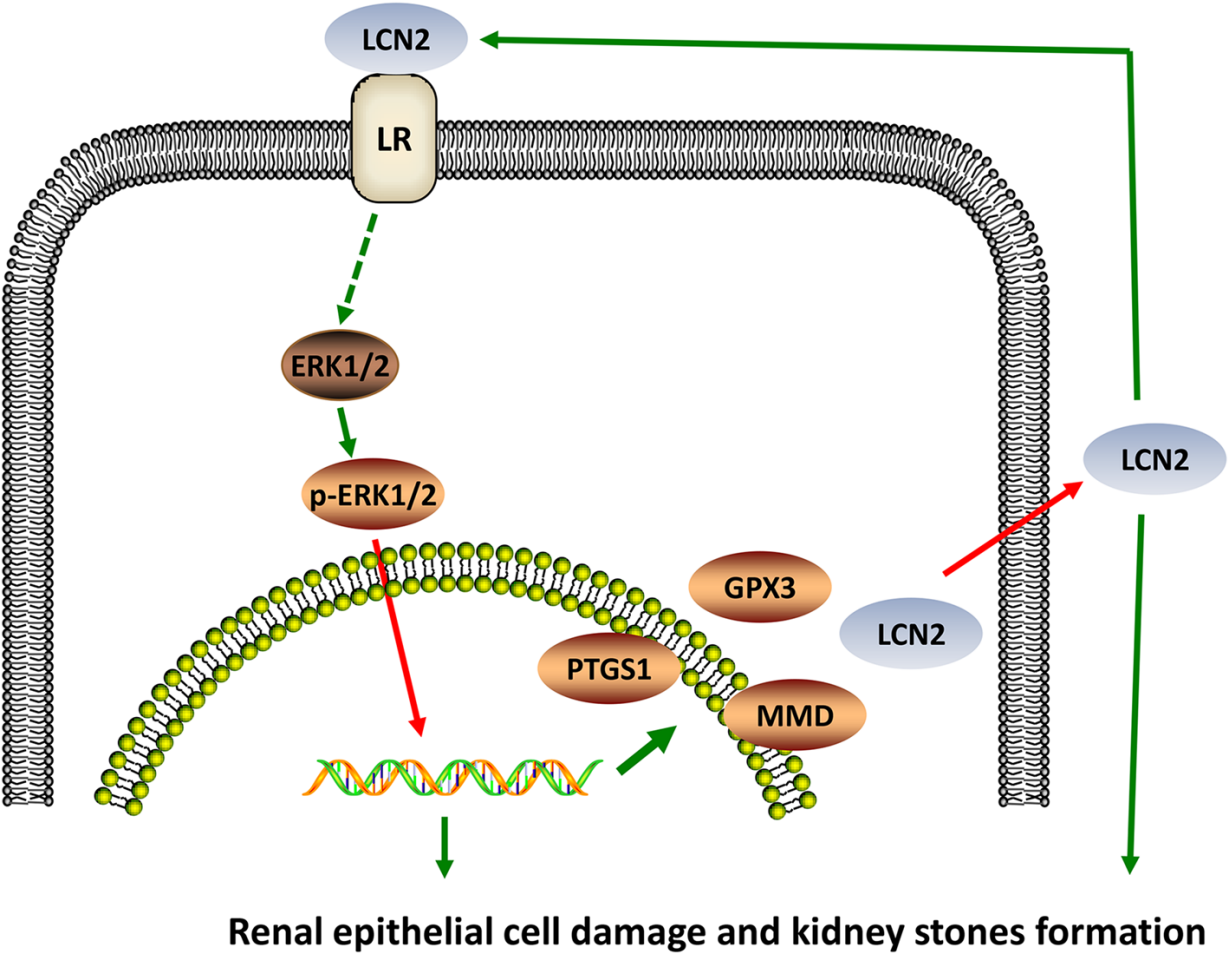
The schematic diagram illustrates the mechanism for the effect of the oxalate/LCN2/ERK and p-ERK and the expressions of LCN2, PTGS1, GPX3 and MMD axises on renal epithelial cell damage during kidney stone formation. Activation of ERK1/2 by oxalate stimulation promotes the expressions of LCN2 and other proteins. Then LCN2, in turn, activates the ERK signaling pathway through its receptor.

In addition to the role of LCN2 in the formation of kidney stones, some studies have proven that many other molecules in kidney stones are differentially expressed^20^. This also implies that they may play a role in the formation and development of kidney stones. In addition, there are studies that refer to the role of certain signalling pathways in the formation of kidney stones^21^. However, the specific molecular mechanisms for the formation of kidney stones so far are still unknown. Based on previous studies we combined signalling molecules with signalling pathways in a double positive circulation regulation model and aimed to further reveal the specific molecular mechanisms of kidney stone formation. A major finding of this study is that LCN2 plays a role in promoting kidney stones through regulation of the ERK signalling pathway and regulation of expression of PTGS1 and other kidney stone-related genes involved in the process of kidney stones. During the formation of kidney stones, the kidney epithelial cells are damaged to promote the accumulation of oxalate crystals^22^^.^ Our results suggest that the overexpression of LCN2 or treatment with rhLCN2 on kidney epithelial cells causes significant cell damage. We also provided the first evidence that the function of LCN2 not only depends on the activation of the ERK signalling pathway but also regulates the activation of the ERK signalling pathway. Thus, LCN2 and the ERK pathway formed a positive feedback loop that may be the reason why we showed that the expression of the relevant molecule fluctuates at an extended point in time. On the one hand, LCN2 regulates the activation of the intracellular signalling pathways and, on the other hand, it regulates the expression of certain kidney stone-related genes, promoting the formation and development of kidney stones from both lateral and longitudinal dimensions (summarized in Fig. 8).

Although the cellular and molecular processes underlying kidney stones have been largely illustrated, the strategies and targets for the treatment of kidney stones are still unsatisfactory. Here, we have examined the regulatory mechanisms, signalling pathway and molecular mechanisms of LCN2 in kidney stones. We revealed a new function of LCN2, as well as its key role in the regulation of ERK signalling during kidney stones upon oxalate stimulation. This study also reveals the relationship between LCN2 and other kidney stone-related genes. An improved understanding of LCN2 might lead to the development of new therapeutic and prognostic markers for kidney stones, thus affecting more patients worldwide.
